# Infliximab for treatment-refractory transverse myelitis following immune therapy and radiation

**DOI:** 10.1186/s40425-018-0471-2

**Published:** 2018-12-22

**Authors:** Victoria A. Chang, Daniel R. Simpson, Gregory A. Daniels, David E. Piccioni

**Affiliations:** 1 School of Medicine, University of California San Diego, La Jolla, California USA; 2 Department of Radiation Medicine and Applied Sciences, University of California San Diego, Moores Cancer Center, La Jolla, California USA; 3 Department of Hematology & Oncology, University of California San Diego, Moores Cancer Center, La Jolla, California USA; 4 Division of Neuro-Oncology, Department of Neurosciences, University of California San Diego, La Jolla, California USA

**Keywords:** Transverse myelitis, Infliximab, Immune-related adverse events, Checkpoint inhibitor, Radiation

## Abstract

**Background:**

Neurologic toxicities with immune therapy are rare, but can cause devastating and often permanent injury when they occur. Although there is increasing interest in the potential synergism between immune therapy and radiation, it is possible that such combinations may lead to a greater number or increased severity of immune-related adverse events. We present here a case of extensive and progressive transverse myelitis following combined therapy, which did not improve until treatment with infliximab. This case highlights the unmet need for treatment of adverse events that are refractory to consensus recommendations, and may ultimately require further study and incorporation into future published guidelines.

**Case presentation:**

We report a case of a 68-year-old with metastatic melanoma, who developed transverse myelitis in the setting of immune checkpoint blockade and spinal irradiation for vertebral metastases. Despite management according to published consensus guidelines: cessation of immune therapy, high-dose steroids, and plasmapheresis, he continued to deteriorate neurologically, and imaging revealed a progressive and ascending transverse myelitis. The patient was then treated with infliximab, and demonstrated dramatic imaging and modest clinical improvement following the first treatment cycle.

**Conclusions:**

This is the first report describing the successful use of infliximab in immune therapy and radiation-related transverse myelitis that was not responding to recommended therapy. Evaluation of additional treatment options such as infliximab for high-grade immune-related neurologic toxicities is warranted, and may be needed earlier in the disease process to prevent significant morbidity. The adverse effects of immune therapy when used in combination with radiation also require further investigation.

## Background

Immune checkpoint inhibitors (ICIs) have revolutionized cancer treatment, producing durable responses in both skin and solid organ malignancies [1]. The currently approved ICIs are monoclonal antibodies targeting the programmed death protein-1 (PD-1) or the cytotoxic-T-lymphocyte-antigen-4 (CTLA-4) [2] pathways which normally limit immune responses. With this shift in the natural balance of the immune system toward its effector arm, immune-related adverse effects are to be expected. Indeed, immune-related toxicities have been demonstrated in nearly every organ system. For severe toxicities (grade 3 or higher according to the Common Terminology Criteria for Adverse Events of the National Cancer Institute), current guidelines suggest management escalation in the following order: ICI cessation, high-dose steroids, other T cell suppressive medications and intravenous immunoglobulin (IVIG) or plasmapheresis [3–5]. In many organ systems, toxicities unresponsive to standard management have also been shown to benefit from immunosuppressive drugs such as tocilizumab and infliximab [6]. Tocilizumab may lead to resolution of ICI-induced cytokine release syndrome [7], arthritis [8], pneumonitis [9], and myocarditis [10]. Infliximab has demonstrated benefit in management of immunotherapy-induced colitis [11, 12] and scleritis [13]. It is unclear if the combination of radiation with ICIs contributes to additional immune related adverse events, although small studies of either systemic or intracranial radiation with ICIs did not report increased toxicity [14, 15].

High-grade central and peripheral nervous system toxicity from ICIs is rare and occurs most commonly in the form of encephalopathies, meningoradiculoneuritis, Guillain-Barre like syndromes, and myasthenic syndromes [16]. Not only are neurologic toxicities rare, but their management when refractory to standard treatment is based on limited reports. Here, we describe a patient who developed transverse myelitis in the setting of ICI therapy and vertebral radiation for metastatic melanoma. His transverse myelitis failed to resolve with standard treatment outlined in published guidelines. In this case report, administration of infliximab produced both clinical and imaging improvement.

## Case presentation

A man in his late 60s with a history of Stage I melanoma of the upper thigh, for which he had undergone wide local excision and negative sentinel lymph node biopsy 2 years prior, presented with new metastatic disease. On imaging, he was found to have lesions of the lung, liver, vertebrae, and brain. Fine needle aspiration of a thoracic lymph node confirmed metastatic melanoma. Next generation sequencing was notable for BRAF V600E mutation.

The patient started treatment with combination ipilimumab and nivolumab. While undergoing immunotherapy, the patient also received radiation to his T7-T10 vertebral metastases (30 Gray (Gy) in 10 fractions) and had stereotactic radiosurgery (SRS) to 16 brain metastases. Spinal irradiation was performed with a 3D conformal technique using opposed anterior-posterior/ posterior-anterior fields. The maximum dose to the spinal canal was 33.5 Gy.

Magnetic resonance imaging (MRI) of the brain following SRS showed marked treatment response. Re-staging computed tomography (CT) of the chest, abdomen, and pelvis, performed 2 months after his initial staging scans, also showed major systemic response. Prior to starting his fourth cycle of ipilimumab and nivolumab, the patient noted the onset of intermittent numbness and tingling of the soles of his feet, with gradual ascension to his knees over the next 2 months.

**Fig. 1:**
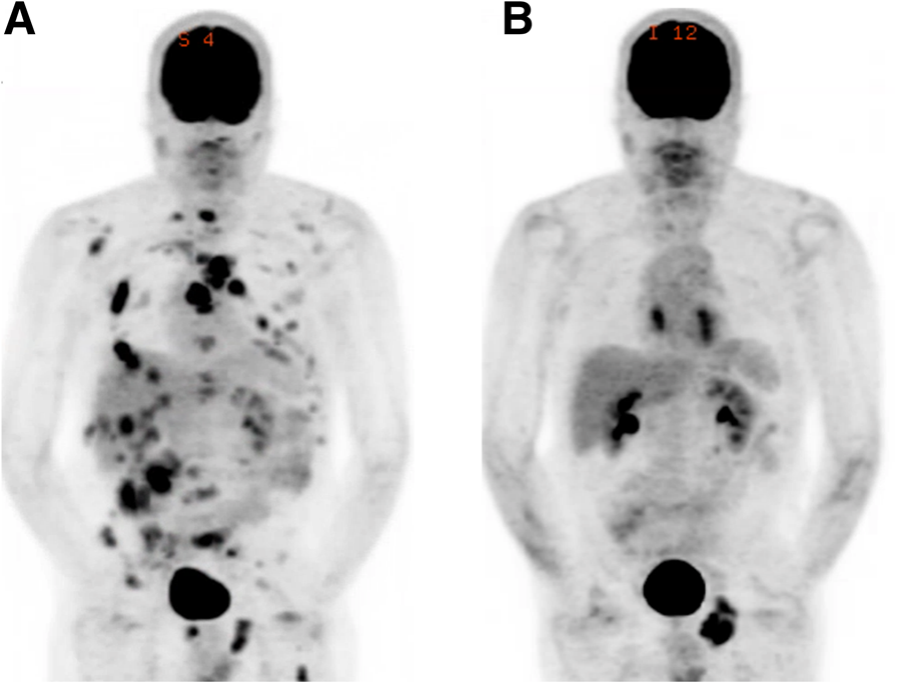
Coronal positron emission tomography images of patient before (**a**) and 5 months after (**b**) initiation of ICI demonstrating complete resolution of widespread hypermetabolic lesions in the lungs, liver, skeleton and mediastinum with the exception of a residual lesion in the left ischium

**Fig. 2:**
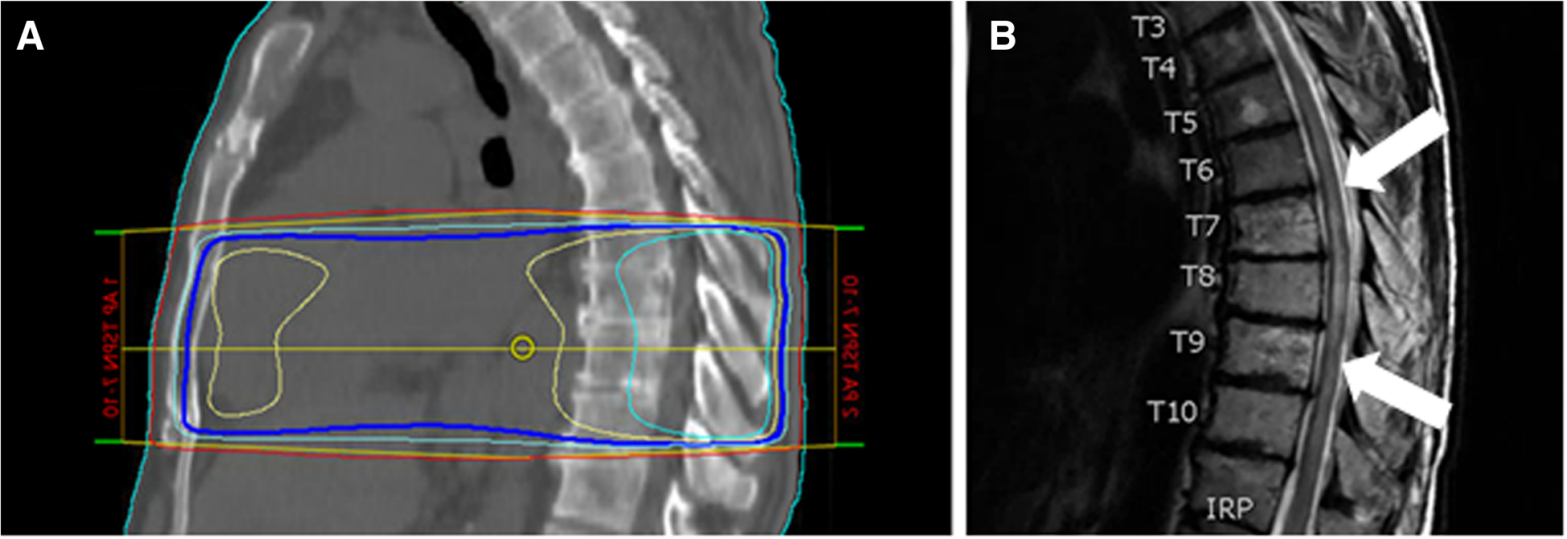
Sagittal images of the (**a**) radiation treatment fields used for palliative spinal radiation extending from thoracic vertebral level T7-T10 and (**b**) follow-up spinal MRI obtained 6 months after radiation therapy showing T2 signal abnormality (white arrows) within the region of previous radiation therapy. The dark blue, yellow, and cyan lines in panel A correspond to the 95, 100, 105% isodose lines, respectively

MRI brain 1 month later showed a new punctate cerebellar metastasis, which was treated using SRS. Positron emission tomography (PET)/CT demonstrated resolution of numerous hyper-metabolic lesions with a remaining area of increased focal uptake in the left ischial tuberosity (Fig. 1). Given evidence of disease progression in the ischial tuberosity but not other systemic areas, the patient transitioned to pembrolizumab and received radiation to his ischial lesion. Approximately 2 weeks after starting pembrolizumab, the patient noted gait instability and ataxia, and further ascension of numbness to the level of his hips. At that time, he was still able to ambulate independently with the assistance of walking sticks. One month after starting pembrolizumab, the patient presented to the emergency department (ED) with 1 day of urinary retention and fecal incontinence. A spinal MRI was performed which showed T2 signal abnormality and patchy enhancement in the thoracic spinal cord (T5 to T10) concerning for myelitis or radiation necrosis without evidence of tumor or malignant cord compression. The T2 signal abnormality corresponded with the thoracic spinal radiation field (Fig. 2). Given that the lesion was enhancing and initially confined to the radiation field, radiation necrosis was favored at that time.

The patient’s immunotherapy was discontinued, steroids (dexamethasone 8 mg twice daily) were initiated, and two doses of bevacizumab (for possible radiation necrosis) were administered, without improvement. Lumbar puncture was deferred due to recent bevacizumab. Given the lack of improvement to optimal therapy for radiation necrosis, transverse myelitis was then favored. Results of serologic evaluation of metabolic (vitamin B12, thyroid stimulating hormone), infectious (human immunodeficiency virus, rapid plasma reagin), and autoimmune (anti-nuclear antibodies, anti-Ro/La, aquaporin-4 immunoglobulin G, erythrocyte sedimentation rate, C-reactive protein) etiologies of transverse myelitis were normal. The patient was trialed on high-dose intravenous methylprednisolone (1000 mg daily for 5 days) for transverse myelitis. His lower extremity numbness and gait instability progressed and he started plasmapheresis.

**Fig. 3:**
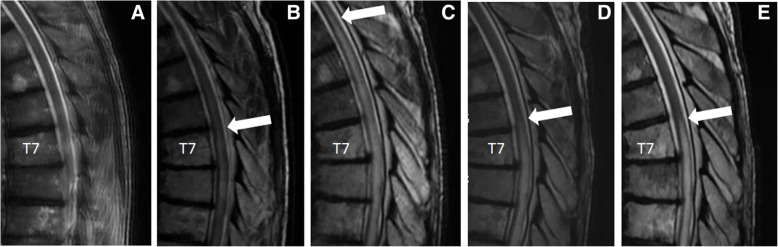
T2-weighted sagittal MRI images of thoracic spine. The 7th thoracic vertebrae is marked for reference. Arrow indicates the superior border of the transverse myelitis. **a** before initiation of therapy, no intrinsic spinal cord lesion. **b** 6 months after radiation, with cord changes around T7. **c** 11 months after radiation, with progressive symptoms and ascending transverse myelitis despite therapy. **d** Three weeks after initiation of infliximab, with significant improvement. **e**. Two months after infliximab, still with some cord abnormality around T7

Following 15 sessions of plamapheresis, a dose of cyclophosphamide 1000 mg/m^2^ was added but the patient continued to decline with worsening urinary retention, bilateral lower extremity spasticity, and complete loss of lower extremity sensation to T5. He did not have upper extremity involvement. Cerebrospinal fluid (CSF) analysis at that time was remarkable for elevated protein (total protein, 99 mg/dL; institutional normal range, 15–45 mg/dL) and negative for malignant cells. Myelin basic protein was elevated at 31.6 ng/mL (normal < 5.5), and oligoclonal bands were matched in the serum and CSF, consistent with an ongoing systemic immune reaction. CSF albumin index was mildly elevated, suggestive of slight impairment of the blood-CSF barrier. Serum studies for antibodies to human T-lymphotropic virus (HTLV) I and II, and a paraneoplastic panel (anti-NR1, anti-GAD65, anti-alpha 3AChR, anti-LGI1, anti-VGCC, anti-VGKC, anti-CASPR2, anti-amphiphysin, anti-CV2, anti-Hu, anti-Ma, anti-Ta, anti-recoverin, anti-Ri, anti-Yo, anti-Zic4) were negative. A serum IL-6 level was normal. A serum TNF-alpha level was not obtained. MRI of the brain demonstrated two new intracranial metastases. MRI of the spine showed progression of transverse myelitis from T3 to T11 (Fig. 3c), now clearly outside the radiation field. Body PET/CT revealed worsening osseous metastatic lesions; therefore the patient began dabrafenib and trametinib. Given his ascending transverse myelitis despite optimal therapy other options including tocilizumab and infliximab were considered. Based on the low IL-6 level, the patient was started on infliximab. Spinal MRI 3 weeks after the first dose of infliximab showed a dramatic reduction of the level of the T2 cord signal abnormality back to T6 to T10 (Fig. 3d) with corresponding improvement in sensory level and muscle spasms. Continued treatment with infliximab led to additional incremental gains on imaging but without further clinical improvement. He subsequently developed systemic progression on dabrafenib and tremetinib (but with stable central nervous system disease) and ultimately succumbed to his disease.

## Discussion and conclusions

We describe a patient with metastatic melanoma who developed progressive transverse myelitis following combination therapy with ICIs and spinal irradiation. His sensory symptoms first manifested approximately 3 months after starting ipilimumab/nivolumab and radiation therapy, and acutely worsened when he was transitioned to pembrolizumab. Our patient was unresponsive to the standard of care recommended by several consensus groups (discontinuation of immunotherapy, high dose steroids, and extensive plasmapheresis treatments), but demonstrated modest clinical and significant imaging improvement with infliximab. To our knowledge, this case represents the first report of successful treatment of ICI-induced transverse myelitis with infliximab. While most cases can be managed with the discontinuation of immunotherapy and steroids, the optimal steroid dosing for transverse myelitis and immunotherapy-related toxicities has not been prospectively defined. Per the American Academy of Neurology guidelines, the recommended dose and duration of steroids for TM is 1 g IV methylprednisolone daily for 3 to 7 days [17]. The National Comprehensive Cancer Network guidelines for immunotherapy-related transverse myelitis suggest at least 2 mg/kg/day of methylprednisolone and to strongly consider 1 g daily for 3–5 days. The next recommended steps for unresponsive cases are IVIG or plasmapheresis. There is no consensus on how to proceed following the failure of plasmapheresis. A recent case of ICI-related encephalitis also showed durable response to infliximab [18]. That case, along with the case presented here, suggest that infliximab may be beneficial for refractory cases of neurologic ICI adverse events.

Causality is particularly challenging to attribute in this case because the patient received immunotherapy and radiation treatment concurrently, and the area of involvement starts within the radiation field. Several features suggest a combined effect of radiation and immune therapy. First, the dose of radiation that this patient received (30 Gy in 10 fractions) is well below the tolerance dose of the spinal cord and highly unlikely to cause myelopathy in isolation [19–21]. Second, the time course of his symptom progression is inconsistent with classically described forms of spinal cord radiation toxicity, which is typically a late complication that develops more than 6 months following RT [22]. Third, the acute worsening of his symptoms 2 weeks after starting pembrolizumab and the extension outside the radiation field further suggests an autoimmune contribution. Lastly, lack of improvement with bevacizumab, which has been shown to be helpful in radiation-induced myelitis, versus response to infliximab is consistent with a component of immune dysregulation.

Thus, the development of a spinal cord lesion of this severity was likely the consequence of the combined impact of RT and immunotherapy. Immune therapy can lead to adverse autoimmune complications, but typically responds to discontinuing the offending agent, steroids and plasmapheresis. The intensity of the reaction in our patient provides caution in applying overlapping therapies of radiation and immune modulation. As more patients receive combined radiation and immunotherapy, clinicians must be vigilant for adverse events, and may need additional therapies such as infliximab early in the disease process for patients not responding to steroids, IVIG or plasmapheresis, in order to prevent significant toxicity.

## Abbreviations

CSF: Cerebrospinal fluid
CT: Computed tomography
CTLA-4: Cytotoxic-T-lymphocyte-antigen-4
ED: Emergency department
Gy: Gray
HTLV: Human T-lymphotropic virus
ICI: Immune checkpoint inhibitor
IVIG: Intravenous immunoglobulin
MRI: Magnetic resonance imaging
PD-1: Programmed death protein-1
PET: Positron emission tomography
SRS: Stereotactic radiosurgery
